# The Impact of Personality Traits on the Outcome of Total Knee Arthroplasty

**DOI:** 10.1155/2016/5282160

**Published:** 2016-02-18

**Authors:** A. Giurea, G. Fraberger, P. Kolbitsch, R. Lass, E. Schneider, B. Kubista, R. Windhager

**Affiliations:** Department of Orthopedics, Medical University of Vienna, Währinger Gürtel 18-20, 1090 Vienna, Austria

## Abstract

Ten to twenty percent of patients with total knee arthroplasty (TKA) are dissatisfied with their clinical outcome. Aim of this study was to investigate the impact of personality traits on the subjective outcome of TKA. We investigated 80 patients with 86 computer navigated TKAs. We asked for patients satisfaction and divided patients into two groups (satisfied or dissatisfied). 12 personality traits were tested by the Freiburg Personality Inventory (FPI-R). Postoperative examination included Knee Society Score (KSS), Western Ontario and McMaster University Osteoarthritis Index (WOMAC), and the Visual Analogue Scale (VAS). Radiologic investigation was done in all patients. 84% of our patients were satisfied, while 16% were not satisfied. The FPI-R showed statistical significant influence of four personality traits on patient satisfaction: life satisfaction (*p* = 0.006), performance orientation (*p* = 0.015), somatic distress (*p* = 0.001), and emotional stability (*p* = 0.002). All clinical scores (VAS, WOMAC, and KSS) showed significantly better results in the satisfied patient. Radiological examination showed optimal alignment of all TKAs. There were no complications requiring revision surgery. The results of our study show that personality traits may influence patients satisfaction and clinical outcome after TKA. Therefore patients personality traits may be a useful predictive factor for postoperative satisfaction after TKA.

## 1. Introduction

Total knee arthroplasty (TKA) is one of the most commonly used procedures for treatment of knee osteoarthritis [1]. Although there have been several improvements in design and implantation technique over the years, there are still a respective number of patients dissatisfied with the clinical outcome after TKA [2–4]. Literature shows a dissatisfaction rate between 10 and 20 percent, although objective examinations cannot show any reasons for dissatisfaction or complications [2–4]. Computer navigated implantation techniques result in improved positioning and alignment of TKAs [5–9] and provide us with a patient cohort with defined alignment of TKAs. Malalignment, range of motion (ROM) [9], patient's age [10], socioeconomic factors [11], and preoperative severity of osteoarthritis [12] are factors influencing patients' satisfaction after TKA but some factors still remain unknown. A former study has shown that the patient's satisfaction following foot surgery may be influenced by individual personality traits [13]. The aim of our study was to investigate if personality traits have an impact on patient's satisfaction and clinical outcome after navigated TKAs.

## 2. Material and Methods

We performed a prospective controlled clinical study. The study received ethical approval from the regional institutional review board (ref. number 382/2011). An informed consent was given by every patient.

### 2.1. Patients and Type of Prosthesis

We included 80 patients who received 86 navigated e.motion® UC (Aesculap AG, Tuttlingen, Germany) knee prostheses between 2009 and 2011. The e.motion® UC endoprosthesis is CE-marked, cruciate retaining knee prosthesis with a rotating polyethylene (PE) and high congruency. Mean age at operation was 66 years (54–81) and gender distribution was 48 females and 32 males. Minimum follow-up period was two years. Only patients without psychic disorder and with no psychopharmacal medication were included. Further inclusion criterions were stability of the TKA and good range of motion (ROM) with a minimum of 100° and the absence of complications.

### 2.2. Surgical Procedure

All surgical procedures have been carried out through a medial parapatellar arthrotomy. Patients were given a perioperative antibiotic prophylaxis with cefazolin (3 × 2 g) or clindamycin (3 × 600 mg). Thrombotic prophylaxis was used with low-molecular weight heparin (40 mg–60 mg/day) starting 12 hours before surgery continuing for 6 weeks postoperatively. A tourniquet was used before osteotomies have been carried out and released after implant fixation. All implants were cemented using gentamycin containing cement (Palacos® R + G, Heraeus, Hanau, Germany) and vacuum cementing technique. The patella was routinely resurfaced and a lateral release was undertaken when necessary to achieve satisfactory patellar tracking. The postoperative management was similar for all patients with the use of crutches for 6 weeks postoperatively.

### 2.3. Navigation

All implantations were carried out by the use of the Orthopilot® (Aesculap AG, Tuttlingen, Germany) navigation system. Navigation was used in order to ensure alignment of the prosthesis in a defined position [5, 7, 8, 14, 15]. For inclusion the TKA has to be implanted in a defined range of alignment in all patients: mechanical femorotibial axis of 0° ± 3° and coronal orientation of the femoral and tibial component of 90° ± 2° and sagittal position of femur component 90° ± 2° and tibia component 90° − 4°.

### 2.4. Clinical Outcome and Patients Satisfaction Measures

To get the actual patients' satisfaction we decided to simply ask by a yes or no question if the patients are satisfied with their TKA or not. Clinical outcome was assessed using the Knee Society Score (KSS, clinical and function scores) and the Western Ontario and McMaster University Osteoarthris Index (WOMAC) [16, 17]. For pain assessment the Visual Analogue Scale (VAS) was used in order to estimate intensity of pain from 1 to 10 [18]. Minimum follow-up period was two years, and none of our patients was suffering from any kind of psychiatric disease, alcohol problems, psychopharmacal medication, or drug addiction. There was no further invasive treatment of the surveyed knee or any obvious complication of the respective knee prosthesis until the time point of follow-up. All TKAs were rated stable and with a minimum range of motion (ROM) of 100°.

### 2.5. Radiological Examination

**Figure 1:**
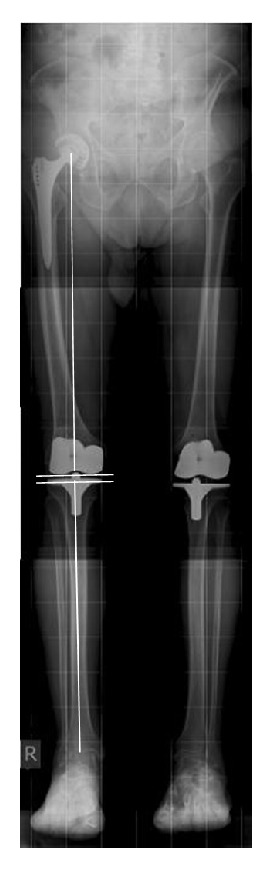
Long leg standing X-ray performed in anterior-posterior (a.p.) view. The mechanical axis, the distal lateral femur angle, and the proximal medial tibial angle of the TKA were measured.

**Figure 2:**
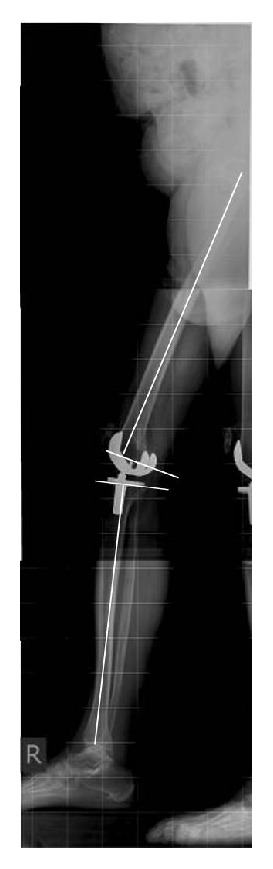
Long leg standing X-rays were performed in lateral view. The femoral flexion/extension angle and the tibial slope of the prosthesis were determined.

Long leg standing X-rays and plain knee radiographs were performed in anterior-posterior (a.p.) and lateral view in all patients and were evaluated by 2 independent observers assessing alignment as well as signs of loosening such as radiolucent lines and osteolyses (Figures 1 and 2). Alignment was measured by the use of integrated radiologic measurement tool Agfa® Impax client (Agfa, Mortsel, Belgium). In the long standing a.p. radiographs the mechanical axis, the distal lateral femur angle, and the proximal medial tibial angle of the TKA were measured. In the lateral long leg radiography the femoral flexion/extension angle and tibial slope of the prosthesis were determined (Figure 2). Radiologic examination was done in all patients to investigate if the defined alignment of TKA implantation was achieved by navigation.

### 2.6. Freiburg Personality Inventory

To investigate the personality parameters we used the Freiburg Personality Inventory-Revised (FPI-R) [13, 19–23]. This multidimensional validated personality form consists of ten traits and two dimensions of personality. Testing is done answering 138 questions by self-evaluation. The scales are life satisfaction, social orientation, performance orientation, inhibition, excitability, aggressiveness, strain, somatic distress, health worries, openness, extraversion, and emotional stability. The norms of the FPI-R are derived from a representative sample of population, which includes 2.035 probands. Higher scores represent higher expression of the items.

### 2.7. Statistical Analysis

To compare scores revealing physical functioning with quality of life, pain intensity, and personality traits we used SPSS 21 for statistical analysis. For describing data we used contingency tables and chi-square distribution. Whenever variances were heterogeneous and data were interval scaled *t*-tests and variance analysis (ANOVA) were used due to the fact that our population was independent. Whenever variances were not heterogeneous rank sum test or Kruskal-Wallis analyses were performed. Level of significance was 95%. Main null hypothesis: there were no significant differences in personality traits in satisfied and dissatisfied participants. Alternative hypothesis: satisfied and dissatisfied participants have different levels of personality traits. Knee society scores and the Western Ontario and McMaster University Osteoarthritis Index (WOMAC) were compared using a *t*-test a variance analysis.

## 3. Results

### 3.1. Patient's Satisfaction and Clinical Outcome

From 86 TKAs 84% (*n* = 72) of patients were declared satisfied with the result, whereas 16% (*n* = 14) were dissatisfied. Preoperative demographic data and preoperative KSS showed now difference between satisfied and dissatisfied patients (Table 1). Satisfied patients showed significantly better clinical outcome postoperatively, although there was no difference in ROM and knee stability between the two groups (Table 2).

Visual Analogue Scale (VAS) in satisfied patients was 1.1 (SD ± 1.5) compared to 6.7 (SD ± 1.8) in dissatisfied patients (*p* < 0.001). WOMAC score in the satisfied group was 0.86 (SD ± 1.3) and 5.76 (SD ± 2.2) in the dissatisfied group (*p* < 0.001). Knee Society Score (KSS) and the Knee Society Function Score were statistically significantly better in satisfied patients (*p* < 0.001) than in dissatisfied patients. The respective scores were 92 (SD ± 13) and 88 (SD ± 16) in the satisfied group and 65 (SD ± 17) and 59 (SD ± 22) in the dissatisfied group (Table 2). ROM showed no difference between satisfied, 118° (SD ± 11.4), and dissatisfied patients, 117° (±18.3) (*p* = 0.262). Both groups had 25 (±0) pts. for stability evaluated from the KSS (Table 2).

### 3.2. Personality Traits

Evaluation of personality traits using the FPI-R revealed statistically significant difference between the two groups in 4 scales (Table 3).

Satisfied patients showed significantly higher scores for life satisfaction (*p* = 0.006) and performance orientation (*p* = 0.015), whereas dissatisfied patients showed significantly higher scores for somatic distress (*p* = 0.001) and emotional instability (*p* = 0.002). With the numbers available no significant differences were found in the other personality traits of the FPI-R (Table 3).

Thus our alternative hypothesis turned out to be confirmed: dissatisfied and satisfied participants showed different levels of personality traits. They were significantly different in performance orientation, life satisfaction, somatic distress, and emotional stability.

### 3.3. Radiologic Results

Radiological results showed that all alignment parameters were within the desired range for exact alignment. The mean mechanical axis was 1.3° varus (SD ± 1.6), the mean LDFA was 90.3° (SD ± 1.3), and mean MPTA was 89.5° (SD ± 1.3). In the sagittal plane mean femoral flexion was 90° (SD ± 1.5) and a mean tibial slope was 88.1° (SD ± 1.5).

### 3.4. Complications and Radiology

There were no obvious clinical complications requiring any revision or intervention of the TKA in both groups. Radiologic examination showed no sign of wear or loosening of the respective TKA in satisfied and dissatisfied patients.

## 4. Discussion

Aim of every TKA is to reach highest possible physical functioning and a reduction of pain. We assumed that successful knee arthroplasty without any obvious complication goes along with patients' satisfaction. Nevertheless the rate of unsatisfied patients after TKA is high and goes up to 20% [2–4]. Therefore we compared satisfied with dissatisfied patients after standardized navigated total knee arthroplasty. Computer navigation provided us with a group of patients with their TKAs implanted in a defined range of coronal and sagittal alignment. As alignment and ROM may play a certain role in patient's satisfaction [9] both groups showed desired alignment and good ROM two years postoperatively.

Satisfied patients showed significantly better clinical outcome, although there was no difference in ROM and knee stability between the two groups. So we conclude that inferior clinical results in the unsatisfied group are due to higher pain scores and lower function. Both groups had to be in a situation with no medical problems after arthroplasty and all patients received the same kind of prosthesis in the same implantation technique and the same postoperative protocol. As malalignment, range of motion [9], patient's age [10], socioeconomic factors [11], and preoperative severity of osteoarthritis [12] are factors influencing patients satisfaction after TKA; some factors still remain unknown. Aim of our study was to determine if psychological aspects affect patients' satisfaction after TKA using the FPI-R, one of the most used standardized personality tests [19]. These aspects were life satisfaction, social orientation, performance orientation, inhibition, excitability, aggressiveness, strain, somatic distress, health worries, openness, extraversion, and emotional stability. The main result was that 4 personality traits had a significant impact on patient's satisfaction. These 4 traits were life satisfaction (*p* = 0.006), performance orientation (*p* = 0.015), somatic distress (*p* = 0.001), and emotional stability (*p* = 0.002). Personality traits are considered to be stable. This means that life events do not harm personality. Previous studies have shown even after amputation stable parameters in personality traits, although their behaviour pattern changed. The finding in our study is consistent with results in different surgical procedures [13]. There is a group of people remaining dissatisfied after successful surgical knee arthroplasty. In our study those patients had in common that they had a low emotional stability, a high level of somatic complains, a low life satisfaction, and a bad performance orientation. To our knowledge there has never been a study describing the influence of personality traits on the patient's satisfaction after total knee arthroplasty before.

With these results our alternative hypothesis turned out to be confirmed: dissatisfied and satisfied participants showed different levels of personality traits. Therefore we recommend already before surgical procedures to focus on emotional stability, somatic complaints, life satisfaction, and performance orientation. Beside age and severity of osteoarthritis and socioeconomic factors we think that personality traits may be useful as predictive factors for postoperative satisfaction after TKA.

## Figures and Tables

**Table 1:** Preoperative data of satisfied and dissatisfied patients.

Preoperative values	Satisfied patients	Dissatisfied patients	*p* value
	(*N* = 72)	(*N* = 14)	
Age at operation	66.2 (±12.1)	62.3 (±11.3)	0.252
BMI	28.6 (±5.7)	28.0 (±4.9)	0.716
Gender distribution	f/m (41/31)	f/m (10/4)	0.318
Knee score, KSS	46 (±15)	52 (±11)	0.164
Function score, KSS	51 (±14)	50 (±16)	0.899

**Table 2:** Clinical results of satisfied and dissatisfied patients.

	Satisfied patients (*N* = 72)	Dissatisfied patients (*N* = 14)	*p* value
VAS	1.1 (±1.5)	6.7 (±1.8)	<0.001
WOMAC	0.86 (±1.3)	5.76 (±2.2)	<0.001
Knee score, KSS	92 (±13)	65 (±17)	<0.001
Function score, KSS	88 (±16)	59 (±22)	<0.001
ROM	118° (±11.4)	117° (±18.3)	0.262
Stability (KSS, max. 25 pts.)	25 (±0)	25 (±0)	1

**Table 3:** Personality traits in satisfied and dissatisfied patients after total knee arthroplasty—as assessed by Freiburg Personality Inventory-Revised (FPI-R).

FPI scales	Satisfied patients (*N* = 72)	Dissatisfied patients (*N* = 14)	*p* value
Life satisfaction	9.5 (±2.3)	7.4 (±3.3)	0.006^*∗*^
Social orientation	7.8 (±2.3)	7.4 (±2.8)	0.56
Performance orientation	8.3 (±2.5)	6.5 (±2.7)	0.015^*∗*^
Inhibition	4.5 (±2.5)	5.5 (±4)	0.23
Excitability	3.8 (±2.9)	4.9 (±2.9)	0.19
Aggressiveness	35 (±2.9)	4.5 (±3.3)	0.27
Strain	4.3 (±3.3)	5.4 (±4.7)	0.3
Somatic distress	3.4 (±2.5)	6.1 (±3.2)	0.001^*∗*^
Health worries	6.8 (±2.7)	7.3 (±2.6)	0.5
Openness	4.5 (±2.5)	4.6 (±2.2)	0.89
Extraversion	7.4 (±2.8)	6.1 (±2.9)	0.1
Emotional stability	4.4 (±3.3)	7.6 (±4)	0.002^*∗*^

^*∗*^A statistically significant difference.
